# Pro- and Anti-inflammatory Biomarkers Responses after Aerobic Training in Heart Transplant Recipients: A Systematic Review and Meta-analysis

**DOI:** 10.2174/011573403X269909240320061952

**Published:** 2024-04-02

**Authors:** Leandro Tolfo Franzoni, Stephanie Bastos da Motta, Gabriel Carvalho, Rochelle Rocha Costa, Mabel Marciela Ahner, Marco Aurélio Lumertz Saffi, Alexandre Araújo Pereira, Adamastor Humberto Pereira, Anderson Donelli da Silveira, Ricardo Stein

**Affiliations:** 1 Programa de Pós-graduação em Ciências da Saúde: Cardiologia e Ciências Cardiovasculares da Universidade Federal do Rio Grande do Sul – Porto Alegre, Brazil;; 2 Grupo de Pesquisa em Cardiologia do Exercício do Hospital de Clínicas de Porto Alegre - Porto Alegre, Brazil;; 3 Universidade de Brasília – UnB – Brasília, DF, Brazil;; 4 Universidade Federal do Rio Grande do Sul, Porto Alegre-RS, Brazil;; 5 Faculdade de Medicina da Universidade Federal do Rio Grande do Sul – Porto Alegre, Brazil

**Keywords:** Transplant, inflammation, biomarkers, aerobic modality, interleukin-6, heart transplant

## Abstract

**Background:**

Physical exercise (PE) may improve plasma concentration of interleukin-6 (IL-6), tumor necrosis factor-alpha (TNF-alpha), and adiponectin (adpN) in heart transplant (HT) patients. However, no consistent data is available on this population.

**Aim:**

Thus, we aimed to conduct a systematic review and meta-analysis on the effects of PE over these pro- and anti-inflammatory biomarkers in HT patients.

**Methods:**

Following the guidelines established by the Preferred Reporting Items for Systematic Reviews and Meta-Analyses (PRISMA) 2020 statement, we conducted a systematic literature search in the PubMed, Cochrane, and Scopus databases. Outcomes included IL-6, TNF-alpha, and adpN. Effect size (ES) was calculated using the standardized mean difference with a 95% confidence interval (CI).

**Results:**

The PE group (aerobic modality) was associated with reduced IL-6 compared to the control group (ES: −0.53; 95% CI: −0.99 to −0.06 pg/mL; *P* = 0.026). However, the PE group did not show a significant effect on TNF-alpha and adpN levels (ES: −0.33; 95% CI: −0.79 to 0.13; *P* = 0.16 and ES: −0.20; 95% CI: −0.70 to 0.30 pg/mL; *P* = 0.444, respectively).

**Conclusion:**

PE is associated with IL-6 reductions, although TNF alpha and adpN did not change after this intervention in HT patients. Therefore, PE is an effective intervention to down-regulate IL-6 in post-HT patients.

## 1 INTRODUCTION

Heart transplant (HT) is one of the recommended treatments for eligible patients with end-stage heart failure, associated with improving prognosis and reducing morbidity in the medium term [1]. Nevertheless, HT individuals are still affected by an inflammatory process, which is not cured with the heart being replaced [2]. Due to surgical advances, there is a survival increase [3]. On the other hand, inflammation remains, possibly caused by the use of immunosuppressant’s [4].

Cytokines such as interleukin-6 (IL-6), tumor necrosis factor-alpha (TNF-alpha), and hormones such as adiponectin (adpN) can directly contribute to endothelial dysfunction by decreasing nitric oxide bioavailability and increasing pro-inflammatory adhesion [5-7]. Endothelial dysfunction is related to coronary allograft vasculopathy and predicts a worse prognosis in HT patients [8]. Furthermore, IL-6 and TNF-alpha are known for their pro-inflammatory action; in contrast, adpN is a hormone with anti-inflammatory function and can even down-regulate the action of pro-inflammatory cytokines [9].

Regular physical exercise (PE) has been shown to reduce plasma concentration of IL-6 TNF-alpha and increase adpN in patients with heart failure, that is, promoting down-regulation of pro-inflammatory cytokines and up-regulation of anti-inflammatory hormones [10, 11]. Previous studies with PE on pro- and anti-inflammatory biomarkers have been developed in post-HT patients, [4, 11, 12] but results do not appear to be consistent, and it is not clear whether there is an anti-inflammatory effect on these individuals [13, 14]. Therefore, this study aimed to conduct a systematic review and meta-analysis on IL-6, TNF-alpha and adpN levels after PE training in post-HT patients.

## 2 METHODS

### 2.1 Study Registration

This systematic review followed the recommendations proposed by Cochrane Collaboration and by Preferred Reporting Items for Systematic Review and Meta-Analysis (PRISMA) statement [15, 16]. This study was previously recorded on the International Prospective Register of Systematic Reviews: PROSPERO (CRD42020214085). The review protocol was not prepared. It's worth noting that the registration methodology, search strategy, data extraction/ risk of bias, as well as data analysis, were based on the study by Reichert *et al.* 2018 [17].

### 2.2 Eligibility Criteria

We included randomized clinical trials that evaluated the effect of PE training, without intensity restriction, on pro- and anti-inflammatory biomarkers in HT individuals over 18 years. Exercise programs were restricted to those who performed supervised sessions in cardiac rehabilitation facilities. No other restriction was made regarding musculoskeletal, metabolic, and respiratory diseases. We considered studies that presented aerobic, resistance, or combined interventions (*i.e.*, aerobic plus resistance exercise), and there was no restriction on exercise types, devices, intensity, session duration, weekly frequency, volume, or rest interval. There was no minimum duration of intervention and no maximum duration of time. Furthermore, we included studies comparing individuals who performed PE training and the control group (active or not). Those studies that did not report significant differences between groups before the intervention period were also included. There was no restriction on the publication date. Only studies published in English, Portuguese, and Spanish were included.

### 2.3 Search Strategies

PubMed, Cochrane, and Scopus were used for searching, the last one being performed in March 2022. Moreover, a manual search of reference lists of studies found in databases was conducted. Abstracts or extended abstracts published from conferences, dissertations, theses, pre-print, or ahead-of-print studies were not included. The following terms were used in combination and/or alone: “Heart transplantation,” “exercise,” and “resistance training.” Boolean operators “OR” and “AND” were used to search databases. Details of the PubMed search are shown in Table **1**. Similar searches using the same keywords were conducted in the other databases.

### 2.4 Study Selection and Data Extraction

Two independent reviewers (M.M.A and S.B.P) evaluated the titles and abstracts of articles found during a database search. A discussion firstly solved any disagreements between the reviewers and then by arbitration of a third reviewer (L.T.F). Subsequently, for selected studies or those in doubt, the same two independent reviewers performed full–text reading, following standard criteria that determined both the inclusion and exclusion of studies. Disagreements between two researchers were resolved by consensus and, if necessary, by a third researcher (L.T.F).

The same two independent reviewers performed data extraction. A standardized form containing information of interest that should be extracted was delivered to each reviewer.

### 2.5 Data Items

The data extracted from studies were publication year, participants' gender, number (number included in analyses), age of participants, peak oxygen consumption (VO2peak) at baseline, body mass, body mass index, time of HT, drug therapy, and group characteristics. Regarding the training, data extracted were modality, intervention duration, weekly frequency, session duration, intensity and numbers of sets, and repetitions if applicable. All values (mean and standard deviation) of outcomes for pro- and anti-inflammatory biomarkers for PE and the control group were extracted pre- and post-intervention (IL-6, TNF-alpha and adiponectin).

### 2.6 Methodological Quality Evaluation (Risk of Bias)

The methodological quality evaluation followed recommendations proposed by the Cochrane Collaboration [16-19]. It was performed considering the following variables: Random sequence generation, allocation concealment, blinding of participants and personnel, blinding of outcome assessment, and incomplete outcome data. Two other independent reviewers (M.M.A and S.B.P) evaluated each criterion, and the studies were classified as high risk (if they did not present the criteria), low risk (if they presented the criteria), or unclear risk (if the criteria were not reported).

### 2.7 Data Analysis

Results are presented as standardized mean differences for absolute values between treatments with a 95% CI. Statistical heterogeneity of treatment effects between studies was evaluated by Cochran’s Q test and I2 inconsistency test; values above 50% indicated high Heterogeneity [17]. The random effects model was applied. Meta-analysis compared pro- and anti-inflammatory biomarkers between PE and the control group. Continuous descriptive variables were presented as mean and standard deviation, while the categorical variables were in relative frequency (%). Values of α ≤ 0.05 were considered statistically significant. All analyses were performed using OpenMeta Analyst Software, version 10.10 [18-21].

### 2.8 Ethics and Dissemination

The systematic review does not require ethics clearance since studies published with non-identifiable data are used.

## 3 RESULTS

### 3.1 Selection and Characteristics of the Studies

**Fig. (1):**
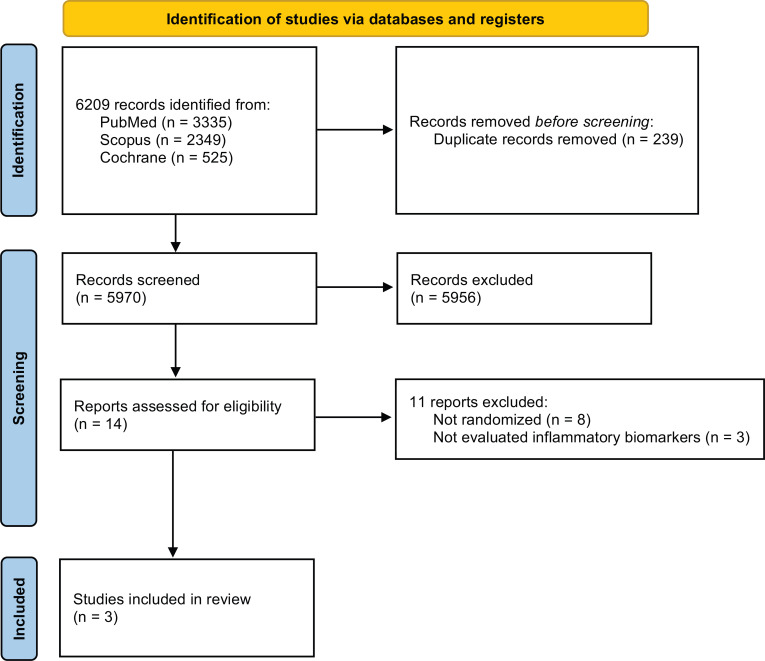
Flowchart of information through the different phases of the systematic review.

Database searches found 6.209 references, being 239 excluded because they were duplicates. After the title and abstract reading, [14] studies met our inclusion criteria for a full-text reading (Fig. **1**). Eight studies were excluded for not being a randomized clinical trial, and three were excluded because they did not evaluate inflammatory biomarkers. For our analysis, we included three studies [4, 12, 13]. Three studies analyzed IL-6, and two of them analyzed TNF-alpha and adpN.

In total, 73 HT participants were included in the meta-analysis. Among these, 38 were in the PE group and 35 in the control group. All studies analyzed both genders (23% women), and the time of heart transplantation was 54.01 ± 44.77 months. Among PE modality, 100% of the intervention group practiced aerobic training (AET), whereas 66.6% (2) used aerobic with high-intensity interval training (HIIT) [4, 13] and 33.3% (1) aerobic with moderated continuous training (MCT) [12]. Among the two studies performing HIIT, one used a stationary bicycle 13, and the other used a stationary bicycle or stair treadmill ergometer [4]. The mean duration of training was 10.67 ± 2.31 weeks (all studies used a training frequency of three sessions per week), and the mean duration of training sessions was 37.33 ± 6.43 minutes. Regarding the control group, only one study used an active intervention group (MCT with stationary bicycle) [13]. Table **2** shows the main characteristics of the included studies.

### 3.2 Risk of Bias

All studies showed an adequate generation of randomization sequence, 33.3% did not report allocation concealment and blinding outcome assessment (1), and 12 and 100% showed a high risk of blinding participants and personnel, as they are studies with PE training, making blinding difficult. Also, 66.6% showed adequate blinding outcome assessment (Tables **3** and **4**) [4, 13].

### 3.3 Physical Exercise *versus* Control Group

**Fig. (2):**
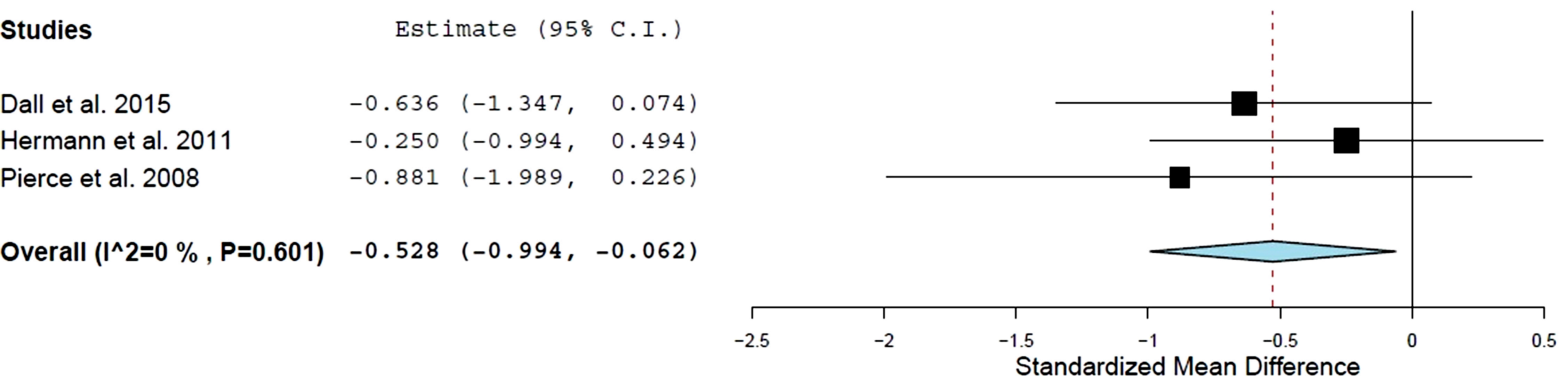
Standard mean differences in interleukin-6 (pg/ml) observed with aerobic training and control group. Filled square: study-specific estimates; filled diamond: pooled estimates of random-effects meta-analysis. **Abbreviations:** Std diff – standardized difference. CI – confidence interval.

**Fig. (3):**
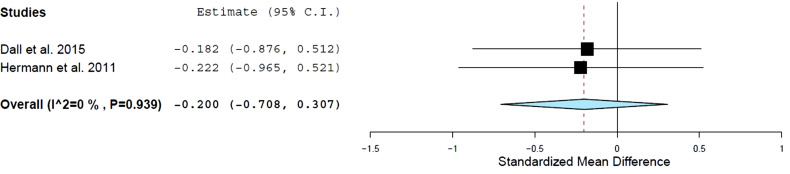
Standard mean differences in tumor necrosis factor – alpha (pg/ml) observed with aerobic training and control group. Filled square: study-specific estimates; filled diamond: pooled estimates of random-effects meta-analysis. **Abbreviations:** Std diff – standardized difference. CI – confidence interval.

IL-6 data were available in three studies, [4, 12, 13]., which compared AET *versus* a control group, with a total of 73 patients evaluated (Fig. **2**). AET was associated with a reduction in IL-6 compared to the control group (effect size: −0.53; 95% CI: −0.99 to −0.06; *P =* 0.03 I2 *=* 0%). This training reduced IL-6 by approximately 0.40 pg/mL compared with the control group (Fig. **3**).

However, TNF-alpha was available in two studies [4, 13] comparing AET *versus* the control group (n=60). Training was not associated with significant changes in TNF-alpha compared to a control group (effect size: −0.33; 95% CI: −0.79 to 0.13; *P* = 0.16; I2 = 0%).

**Fig. (4):**
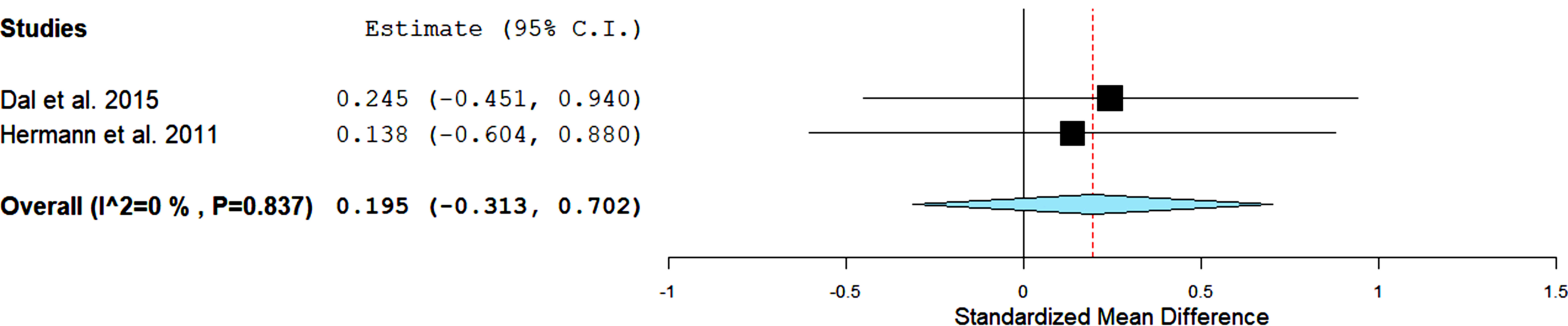
Standard mean differences in adiponectin (pg/ml) observed with aerobic training and control group. Filled square: study-specific estimates; filled diamond: pooled estimates of random-effects meta-analysis. **Abbreviations:** Std diff – standardized difference. CI – confidence interval.

Therefore, adpN data were evaluated in two studies, [4, 13] comparing the effect of AET *versus* the control group, with 60 individuals in the analysis (Fig. **4**). AET was not associated with changes in adpN compared to a control group (effect size: 0.19; 95% CI: -0.31 to 0.70; *P* = 0.44; *I2* = 0%).

## 4 DISCUSSION

To the best of our knowledge, this is the first systematic review and meta-analysis of a randomized clinical trial to investigate the effects of PE training in post-HT patients on important pro- and anti-inflammatory biomarkers, such as IL-6, TNF-alfa and adpN. Our main result in a very selected population demonstrated that PE training (aerobic modality) could significantly reduce IL-6 compared to a control group; in contrast, the same did not occur in TNF-alpha and adpN.

Among the studies evaluated, those that met the inclusion criteria for the pro- and anti-inflammatory biomarkers were only with the AET modality, either HIIT [4, 13] or MCT [12] as an intensity. Interestingly, in one of the trials that used HIIT as an intervention, [13] used MCT as a control. Recently, a systematic review with meta-analysis was published about the HIIT effects on aerobic capacity, demonstrating was more effective compared to MCT, but did not evaluate pro- and anti-inflammatory biomarkers [14]. In HT patients, one difficulty when prescribing AET, HIIT, or MCT may be reaching the heart rate zones predicted by the cardiopulmonary exercise testing (or other exercise testing) due to cardiac denervation and inadequate chronotropic response [19]. However, two studies [4, 13] included patients with an average transplant time of approximately six years or more, and only one [12] included recent HT patients (<12 weeks). After one year of transplantation, reinnervation may occur, facilitating autonomic modulation of heart rate [20].

Regarding IL-6, we know that this pro-inflammatory cytokine plays a fundamental role in immune and inflammatory response, being one of the major mediators of the acute phase of inflammation [21]. AET exerts immunoprotection and an immunoregulatory effect, improving the balance between pro- and anti-inflammatory cytokines [22]. Exercise induces different immune responses associated with the production of interleukins in different populations [23, 24]. Studies suggest that exercise can activate genes involved in leukocyte production and downregulate inflammation [25]. Steenberg *et al*. 2001,[26]. published the first study about the relationship between exercise muscle contraction and reduction in plasma IL-6 levels. Different parameters can modulate IL-6, especially the intensity and duration of PE [27].

Additionally, the noted decrease in IL-6 levels following PE in HT recipients necessitates a thorough evaluation of its clinical ramifications. Elevated IL-6 has been associated with heightened risks of allograft rejection and graft vasculopathy.Consequently, the exercise-induced reduction in IL-6 introduces compelling possibilities for alleviating these complications in individuals who have undergone heart transplantation.

On the other hand, no significant differences were found for TNF-alpha. The measurements had a high variance, which can directly affect the result. If we look at these studies individually, Dall *et al*., 2015 13 observed a reduction of 0.40 pg/mL after an intervention with PE, however, without significant differences. In comparison, the control group showed an increase of 0.40 pg/mL without significant differences. Hermann *et al.,* 2011 [4] observed a reduction of 0.16 pg/mL for the PE group and an increase of 0.37 pg/mL for control, without significant differences. Furthermore, Pierce *et al.,* 2008 [12]. showed that the AET group did not change, but there was a significant increase in the control (1.66 ± 1.02 *vs.* 3.07 ± 1.10 pg/ml, *P*<0.05). It is crucial to emphasize that Pierce *et al*., 2008 exclusively provide absolute data, rendering it unfeasible for inclusion in the meta-analysis. Although the studies did not find significant numerical differences, it is noteworthy that there is a tendency to reduce or maintain values in the PE group, and the increased values after follow-up for the control group may be a good indicator of the potential role of PE in positively reducing TNF-alpha [28-32].

AET did not adpN significantly, with only [4, 13] two studies presenting this anti-inflammatory biomarker. The studies showed opposite results; one of them found reductions in plasma adpN concentrations after follow-up, both for AET and control group, [4] while the other one found an increase in plasma adpN concentrations after follow-up for both groups, [13] with a delta of 23.90 pg/mL for the control group. There is an inverted J-shaped association between adpN and the risk of developing heart failure in adult men [33]. Furthermore, low levels of circulating adpN are associated with diabetes, obesity, and coronary artery disease, as well as an increased risk of acute myocardial infarction, where high levels seem to reduce overall cardiovascular risk [34]. The adpN hormone can also act as an anti-inflammatory and vasculoprotective cytokine [35]. The impact of PE training in adpN in HT patients is still unclear, as the results do not point in the same direction in this analysis.

The high heterogeneity about the time of transplantation and the small number of studies are important limitations. Moreover, the comparison between the PE group (HIIT) and the control active group (*i.e.*, a control group that performed aerobic with MCT) can be a limitation, as well [13, 36]. Finally, we can say that different AET intensity within the same analysis is a limitation. However, we cannot do a sub-analysis for each intensity since we will not have enough studies.

For clinical practice, it is important to carry out studies to obtain answers about the effects not only of AET but also in other exercise modalities, such as resistance or combined training on inflammatory cytokines and hormones in post-HT patients, considering that these modalities can also improve different outcomes in this population.

Finally, these data can be useful, as they add information about the effect of AET on important pro- and anti-inflammatory biomarkers in post-HT patients. In addition, it may serve for future studies to improve methodological aspects, comparing transplanted patients randomized to AET *versus* those without exercise or those who perform another type of exercise.

## CONCLUSION

AET seems to be associated with a reduction in IL-6, an effective intervention to modulate this pro-inflammatory cytokine positively. On the other hand, this training modality did not have the same effect on TNF-alpha and adpN. Since the number of randomized clinical trials in this area of knowledge is small, more well-designed studies are needed to fill this gap.

## DISCLOSURE STATEMENT

This article has previously been published in: “Non-invasive hemodynamics, molecular biology and inflammatory markers from the perspective of physical exercise: from heart failure to heart transplant.” available at: https://lume.ufrgs.br/handle/10183/239008, as part of the lead author’s doctoral thesis.37.

## Figures and Tables

**Table 1:** Search strategy.

**Search**	**Query**
#1	Search ((“Heart Transplantation” OR “Grafting, Heart” OR “Graftings, Heart” OR “Heart Grafting” OR “Heart Graftings” OR “Transplantation, Heart” OR “Heart Transplantations” OR “Transplantations, Heart” OR “Cardiac Transplantation” OR “Cardiac Transplantations” OR “Transplantations, Cardiac” OR “Transplantation, Cardiac”) AND (Exercise OR Exercises OR “Physical Activity” OR “Activities, Physical” OR “Activity, Physical” OR “Physical Activities” OR “Exercise, Physical” OR “Exercises, Physical” OR “Physical Exercise” OR “Physical Exercises” OR “Acute Exercise” OR “Acute Exercises” OR “Exercise, Acute” OR “Exercises, Acute” OR “Exercise, Isometric” OR “Exercises, Isometric” OR “Isometric Exercises” OR “Isometric Exercise” OR “Exercise, Aerobic” OR “Aerobic Exercise” OR “Aerobic Exercises” OR “Exercises, Aerobic” OR “Exercise Training” OR “Exercise Trainings” OR “Training, Exercise” OR “Trainings, Exercise” OR “Resistance Training” OR “Training, Resistance” OR “Strength Training” OR “Training, Strength” OR “Weight-Lifting Strengthening Program” OR “Strengthening Program, Weight-Lifting” OR “Strengthening Programs, Weight-Lifting” OR “Weight Lifting Strengthening Program” OR “Weight-Lifting Strengthening Programs” OR “Weight-Lifting Exercise Program” OR “Exercise Program, Weight-Lifting” OR “Exercise Programs, Weight-Lifting” OR “Weight Lifting Exercise Program” OR “Weight-Lifting Exercise Programs” OR “Weight-Bearing Strengthening Program” OR “Weight-Bearing Strengthening Program” OR “Strengthening Program, Weight-Bearing” OR “Strengthening Programs, Weight-Bearing” OR “Weight Bearing Strengthening Program” OR “Weight-Bearing Strengthening Programs” OR “Weight-Bearing Exercise Program” OR “Exercise Program, Weight-Bearing” OR “Exercise Programs, Weight-Bearing” OR “Weight Bearing Exercise Program” OR “Weight-Bearing Exercise Programs”)

**Table 2:** Characteristics of studies included in meta-analysis.

**Study**	**Participants**	**Participants Number**	**HT Time**	**Intervention**	**Comparator**	**Progression**	**Set**	**Repetitions**	**Intensity**	**Frequency**	**Follow Up**	**Duration**
Pierce *et al.,* 2008	HT patients at least 8 weeks	AET: 8 CG: 6 (over 50 years old)	AET: 68.3 days CG: 78.3 days	Treadmill aerobic exercise (MCT)	Usual care, encouraged to perform physical activity, not supervised exercise	First 4 weeks: 30 min with BORG 11-13, after this, 35-40 min with BORG 12-14	x	x	BORG 11-14	3x	12	30-40 min
Hermann *et al.,* 2011	HT patients at least 12 months	AET: 14 CG: 13 (over 45 years old)	AET: 6.8 ± 4.0 years CG: 7.0 ± 5.5 years	Ergometric bicycle or Staircase running (HIIT)	Patients received classes about nutrition and exercise	x	x	x	> 80 VO_2_peak	3x	8	52 mi
Dall *et al.,* 2015	HT patients at least 12 months	AET: 16 CG: 16 (over 50 years old)	6.4 (1 to 17) years	Ergometric bicycle (HIIT)	Ergometric bicycle (MCT)	x	2 x 4 min 2 x 2 min 4 x 1 min	x	> 80 VO_2_peak	3	12	30 min

**Note:** HT: heart transplant; AET: aerobic training; CG: control group; MCT: moderate continuous training; HIIT: high intensity interval training; BORG: perceived of exertion scale; VO2peak: oxygen consumption.

**Table 3:** Drug therapy.

-	**Pierce *et al*. 2008**	**Hermann *et al.* 2011**	**Dall *et al.* 2015**
Drug Therapy	Tacrolimus; Prednisone; Mycophenolate Mofetil; Valganciclovir; HMG-CoA reductase inhibitor; ACEI/ARB; Insulin; Calcium Channel Blockers; Cyclosporine.	Corticosteroid; Cyclosporine; Tacrolimus; Diuretic; Antihypertensive; ECA inhibitors; Angiotensin 2 blockers; Beta-Blockers; Dihydropyridines; Insulin; Metformin; Statin.	Cyclosporine; Tacrolimus; Mycophenolate; Mofetil; Everolimo; Prednisone; Antihypertensive.

**Note:** HMG-CoA: hydroxymethylglutaryl-CoenzymeA; ACEI/ARB: angiotensin-converting enzyme; ECA: angiotensin-converting enzyme.

**Table 4:** Risk of bias.

**Study**	**Random Sequence Generation**	**Allocation Concealment**	**Blinding of ** **Participants/Personnel**	**Blinding of Outcome ** **Assessment**	**Incomplete Outcome Data**
Pierce *et al*., 2008	Low	Unclear	High	Unclear	Low
Hermann *et al.,* 2011	Low	Low	High	Low	Low
Dall *et al*., 2015	Low	Low	High	Low	Low

## Data Availability

The authors confirm that the data supporting the findings of this research are available within the article.

## LIST OF ABBREVIATIONS

PE: Physical exercise
IL-6: Interleukin-6
HT: Heart Transplant
ES: Effect Size

## STANDARDS OF REPORTING

PRISMA guidelines and methodology were followed.

## SUPPLEMENTARY MATERIAL

PRISMA checklist is available as supplementary material on the publisher’s website along with the published article.
